# Conductance fluctuations in high mobility monolayer graphene: Nonergodicity, lack of determinism and chaotic behavior

**DOI:** 10.1038/srep33118

**Published:** 2016-09-09

**Authors:** C. R. da Cunha, M. Mineharu, M. Matsunaga, N. Matsumoto, C. Chuang, Y. Ochiai, G.-H. Kim, K. Watanabe, T. Taniguchi, D. K. Ferry, N. Aoki

**Affiliations:** 1 Graduate School of Advanced Integration Science, Chiba University, Chiba, 263-8522, Japan; 2 School of Electronic and Electrical Engineering and Sungkyunkwan Advanced Institute of Nanotechnology (SAINT) Sungkyunkwan University, Suwon-Si, South Korea; 3 Advanced Materials Laboratory, National Institute for Materials Science, 1-1 Namiki, Tsukuba, 305-0044, Japan; 4 School of Electrical, Computer and Energy Engineering, Arizona State University, Tempe, AZ, 85287, US

## Abstract

We have fabricated a high mobility device, composed of a monolayer graphene flake sandwiched between two sheets of hexagonal boron nitride. Conductance fluctuations as functions of a back gate voltage and magnetic field were obtained to check for ergodicity. Non-linear dynamics concepts were used to study the nature of these fluctuations. The distribution of eigenvalues was estimated from the conductance fluctuations with Gaussian kernels and it indicates that the carrier motion is chaotic at low temperatures. We argue that a two-phase dynamical fluid model best describes the transport in this system and can be used to explain the violation of the so-called *ergodic hypothesis* found in graphene.

Graphene exhibits a high electron mobility even at room temperature, reaching values as high as 6000 cm^2^/V · s^1^,^2^,^3^,^4^,^5^. Given its weak dependence on temperature, defect scattering seems to be the dominant process limiting the conductivity in graphene^6^,^7^,^8^,^9^,^10^. Indeed, many reports suggest the adsorption of water and oxygen molecules on its surface and special architectures have been proposed to protect it such as sandwiching it with hexagonal boron nitride (*h*-BN) sheets^11^.

Conductance fluctuations (CF) that appear as a function of an external parameter have been used as a probe of the electron dynamics in mesoscopic devices. One key aspect of CF is the so called *ergodic hypothesis* that asserts that the average CF over any external parameter and the average over the statistical ensemble are equivalent. Although this hypothesis implies that the CF are universal in amplitude and independent of extensive parameters^12^,^13^,^14^, it has been shown that in many cases, CF in graphene violate this hypothesis^15^,^16^.

Here, we apply ideas of non-linear dynamics to study the conductance fluctuations in *h*-BN protected graphene (Fig. 1). First, general properties such as the mobility and the mean free path for this material are obtained from Shubnikov - de Haas oscillations. We then study CF as a function of the back gate voltage and magnetic field to check for ergodic behavior. Furthermore, the power spectra of these fluctuations is calculated and a simple model based on harmonic oscillators is used to estimate the density of defects and compare it to that of a standard graphene sample deposited on a SiO_2_ substrate.

By applying the semi-classical theory to the hierarchical phase spaces of mixed chaotic systems, it has been demonstrated that phase coherent transport in chaotic structures with a mixed phase space and a power-law escape from the structure generically produces fractal CF^17^,^18^. Here we compute the Haussdorff dimension and the *determinism* (see methods section) of the CF to check for this fractal signature and analyze the nature of the potential landscape of this graphene device. Finally, an estimate for the distribution of eigenvalues is determined from the fluctuations using Gaussian kernels and a localization study of the magnetoconductance is performed.

We conclude by arguing that a two-phase dynamical fluid model consisting of a stochastic quantum mechanical phase and a deterministic classical phase can be used to explain the violation of the *ergodic hypothesis*.

## Results

The carrier concentration estimated by Shubnikov-de Haas (SdH) measurements at 0.3 K changes linearly with the applied gate voltage by approximately 6.45 × 10^10^ cm^−2^/V for electrons and 6.77 × 10^10^ cm^−2^/V for holes. The maximum mobility is approximately 1.16 × 10^5^ cm^2^/V · s for holes and 9.62 × 10^4^ cm^2^/V · s for electrons computed by sweeping the back gate voltage (supplementary material Fig. S1). The maximum mean free path is also calculated by sweeping the back gate voltage and is approximately 420 nm independent of the carrier type. The charge neutrality point is at −2.09 V. All SdH measurements showed a Berry phase around *π* indicating the presence of Dirac fermions, in agreement with previous results^19^. A fan diagram of the SdH oscillations together with a plot of the Berry phase as a function of the back gate voltage are shown in the supplementary Fig. S2.

The conductance fluctuations (Δ*G*, CF) shown in Fig. 2b were calculated from the conduction curves found in Fig. 2a by subtracting a simple moving average with a window of 0.6 V. The amplitude of the CF is not constant, and oscillates by ~24% between 0.72 to 0.55 *G*_0_ (G_0_ is the quantum of conductance: 4*e*^2^/*h*, where *e* is the fundamental electric charge and *h* is Planck’s constant).

Log-log plots of the root mean square (RMS) of the fluctuations for both the transconductance and the magnetoconductance curves are shown in Fig. 2e. The curves show two distinct regions with a crossover around 3 K. One region is characterized by a T^−1/2^ dependence, whereas the other one shows saturations at values smaller than the expected theoretical maximum (~0.35 *G*_0_)^15^.

The power spectral density (periodogram) of the fluctuations was compared to that of the fluctuations obtained from a typical graphene sample deposited on a standard SiO_2_ substrate. As Fig. 3 indicates, the power distribution density for the latter is more peaked and has its maximum at around 0.25 V^−1^ for the P region. The maximum for the graphene sample sandwiched between BN sheets is at around 0.93 V^−1^ for the N region and 0.66 V^−1^ for the P region. On the average, the spectra for the fluctuations in the N region is approximately 1.78 times more intense than that in the P region.

Figure 2c,d shows the magnetoconductance fluctuations obtained by sweeping a magnetic field perpendicular to the sample. The same procedure of removing the low-frequency background was used to obtain the fluctuations. Above approximately 0.3 T, Shubnikov-de Haas oscillations become prominent. These fluctuations are one order of magnitude smaller than those obtained by sweeping the Fermi level.

The Hausdorff dimension, obtained for changes in gate voltage smaller than ~295 mV, is shown in Fig. 4a. *Determinism* is a measure of complexity that can be used to quantitatively estimate the predictability of a signal. We use this measure to check how random or periodic a fluctuation is. The determinisms, considering the Fermi level sweep for the N and P regions shown in Fig. 4b, are statistically equivalent down to approximately 10 K. For lower temperatures, however, the determinism for the CF of the N region drops much faster than that for the P region. From this, we can estimate an activation energy near 10 · *k*_*B*_ (~0.86 meV).

It has been postulated that the spectra of time-reversal invariant systems whose classical counterparts show chaotic behavior should have the same fluctuation properties of a Gaussian orthogonal ensemble (GOE) or a Gaussian symplectic ensemble (GSE)^20^,^21^. When the system does not exhibit time reversal symmetry, a Gaussian unitary ensemble (GUE) better models it^22^. The distributions of the eigenvalue spacing were estimated as a probability density function as shown in Fig. 5, together with the theoretical distributions (the evolution of the distributions at different temperatures is shown in supplementary Fig. S3). A Kolmogorov-Smirnov (K-S) test^23^,^24^ was performed to check which probability density function better fits the experimental data and the results are given in Fig. 6. At lower temperatures the distributions for both the P and N regions tend towards a GOE.

Magnetoresistance curves were taken at different back gate voltages between −1 T and 1 T as shown in Fig. 7. The peaks at zero magnetic field were analyzed and the heights of the peaks are plotted as a function of the distance from the charge neutrality point for the P and N regions. This is not a pure weak localization peak, but rather it includes a geometrical effect given by the arrangement of the contacts.

## Discussion

Assuming that the disorder potential is smooth enough so that it could be modeled by a harmonic confinement and that peaks in the conductance fluctuations correlate to peaks in the density of states, it should be possible to associate the main peak of the Fourier transform of the conductance fluctuations (*ω*_0_) to the mean spacing between the eigenvalues of the system. Thus, it should be possible to estimate the average depth of the confinement potential as *ħω*_0_/*q*.

According to this model, and the obtained power spectra, the average potential felt by the n-type carriers is only half of that felt by the p-type carriers. Furthermore, from the maximum radius of the confinement potential it is possible to estimate that the density of scattering centers for a graphene flake sandwiched between *h*-BN sheets is approximately 3.6 times smaller than that for a graphene flake exfoliated on a SiO_2_ substrate. Furthermore, the broader spectra found in the former suggests that the spatial separations between scattering centers may be more distributed.

The amplitude of the conductance fluctuations obtained by sweeping the magnetic field is approximately ten times smaller than those obtained by sweeping the Fermi level, which is the result of a non-ergodic behavior found in graphene^15^.

Although the correlation radius obtained from the magnetoconductance fluctuations is around 110 nm between 0.3 K and 44 K, the resistance fluctuation amplitude is proportional to the thermal length 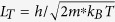 down to a point between 3 K and 10 K. As the temperature is lowered, the thermal length exceeds the size of the sample (for instance, *L*_*T*_ = ~1.4 *μ*m at 10 K) and saturation occurs in agreement with previous results^25^,^26^. Measurements below this threshold are not affected by finite size effects. This behavior is typically more difficult to observe in graphene over SiO_2_, since the crossover temperature is affected by the density of scatterers.

The determinism found at 0.3 K for the sample deposited on a SiO_2_ substrate was 96.7%. Although the determinism for our sample is above 80% at high temperatures, it quickly drops for temperatures lower than approximately 10 K. Hence, *h*-BN sandwiched graphene appears to have a lower density of scatterers, and this makes it possible to observe manifestations of a chaotic behavior.

The progressive increase of the Hausdorff dimension for low temperatures indicates that the conductance fluctuations are not purely random. Rather, they have a statistical fractal structure associated with an increase in the complexity of the mechanisms that create them. Above approximately 10 K, the Lebesgue measure of the fluctuations is zero, which indicates a very simple, nearly binary, mechanism associated with the fluctuations. On the other hand, for lower temperatures, the fluctuations become more complex, assuming a variety of values that approach the Hausdorff dimension of a pure Wiener process (*H* = 1.5).

The progressive reduction in the K-S statistic as the temperature is lowered and the fractional Hausdorff dimension found indicate that one effect of the scattering centers is to produce a chaotic motion of the carriers. It is important to point out that the kernel of the conductance fluctuations obtained for graphene deposited on a SiO_2_ substrate can also be fitted with a GOE distribution. Although preparing a graphene sample sandwiched with *h*-BN sheets increases the carrier mobility, the possible chaotic motion is unaffected.

The time it takes for the width of a propagating coherent state in an open chaotic system to grow from the Fermi wavelength to the linear size of the system is called Ehrenfest time. If the time of flight across the system is much shorter than the Ehrenfest time, then wave packets have no time to diffract and one can expect a suppression of stochastic quantum mechanical channels in favor of deterministic channels mediating the transport. This has interesting manifestations such as the elimination of shot noise^27^.

If, however, the ratio between these time scales is finite, then a two-phase dynamical fluid with a stochastic quantum mechanical phase and a deterministic classical phase mediates the transport. Moreover, when the two time scales are comparable, deviations from the universal behavior are expected^28^. Given the high mobility found in graphene, this situation could be expected.

Given that our system is chaotic and we see no clear evidence for weak localization, we argue that the violation of the ergodic hypothesis in graphene can be described by the two-phase dynamical fluid model. Furthermore, the fluctuations are a direct consequence of long diffracting orbits, and these are much more sensitive to the external magnetic field, thus producing bigger conductance oscillations.

In summary, the violation of the ergodic hypothesis in graphene is not due to a finite size effect. The charge transport that produces conductance fluctuations is chaotic, and its manifestations can be seen in clean systems such as the one composed of a graphene flake sandwiched between *h*-BN sheets. Such systems are open quantum chaotic systems and we argue that, as such, the transport can be mediated by a two-phase dynamical fluid model that can violate the ergodic hypothesis.

## Methods

### Sample Preparation

**Figure 1:**
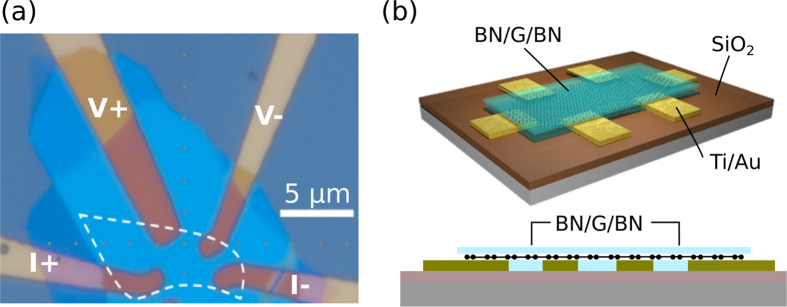
(**a**) Optical microscopy image of the sample with indicated current (I) and voltage (V) electrodes. The dotted white line indicates the position of the graphene flake and the light blue regions are the top and bottom *h*-BN sheets. (**b**) Schematic of the *h*-BN encapsulated graphene (BN/G/BN) device (top) and its cross-sectional view (bottom).

**Figure 2:**
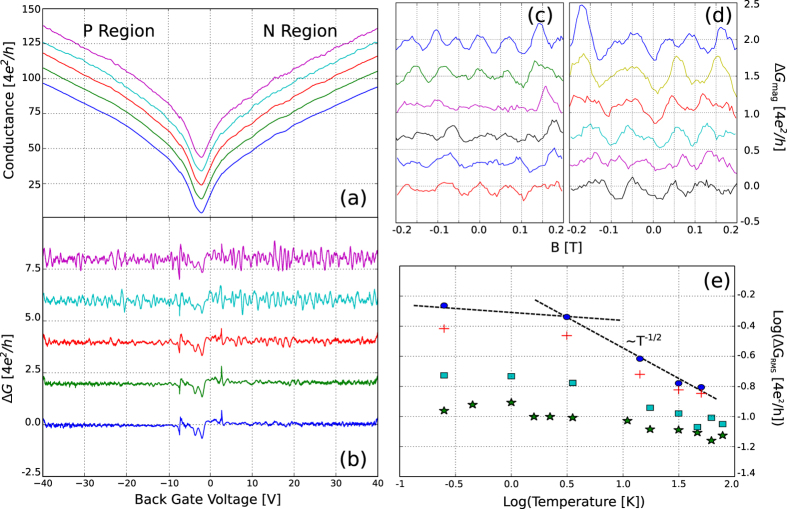
(**a**) Conductance of the graphene flake taken at different temperatures. N and P regions are those related to voltages higher and lower than the charge neutrality point, respectively. (**b**) Conductance fluctuations obtained from the conductance curves. From top to bottom: 0.3 K, 2.67 K, 10 K, 20 K and 30 K. (**c**,**d**) Magnetoconductance fluctuations obtained with back gate voltages −5 V and +5 V measured from the charge neutrality point. From top to bottom: 0.3 K, 3.0 K, 12 K, 20 K, 28 K and 36 K. Offsets were added for better visualization. (**e**) Log-log plots of the RMS value of the fluctuations for p-type (green 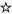) and n-type (cyan 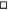) magnetoconductance fluctuations and p-type (red +) and n-type (blue 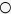) transconductance fluctuations. Dashed lines indicate two distinct regions: a region with a T^−1/2^ dependence and a saturation region.

**Figure 3:**
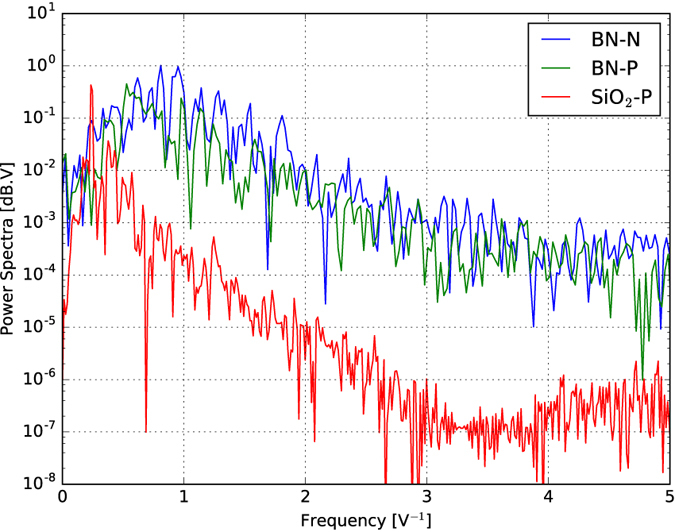
Logarithm of the power spectra (periodogram) of the conduction fluctuations for a graphene sample sandwiched between two boron nitride sheets (top blue curve for the N region and top green curve for the P region) and another graphene sample deposited on a SiO_2_ substrate (bottom red curve). Both curves were obtained at 0.3 K.

**Figure 4:**
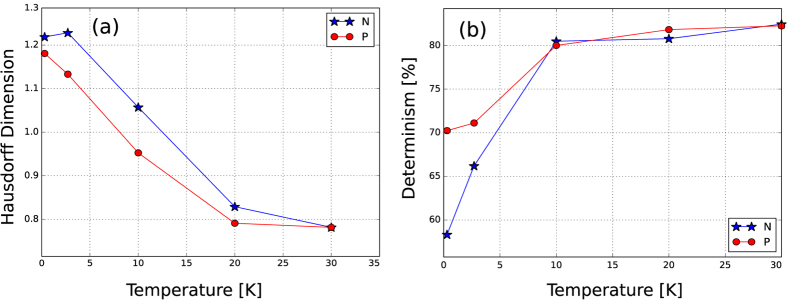
(**a**) Hausdorff dimension and (**b**) determinism as a function of temperature for the P (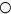) and N (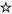) regions. As a comparison, for a typical graphene sample fabricated on a SiO_2_ substrate in our group, the Hausdorff dimension is ~1.3 and the determinism is ~97% at 0.3 K.

**Figure 5:**
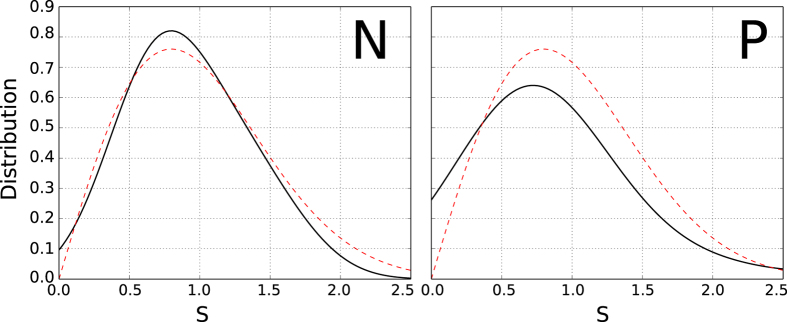
Theoretical GOE (red dashed curves) and the estimated probability density functions (solid curves) for the N (left) and P regions (right) as a function of the spacing ‘*s*’ obtained at 0.3 K.

**Figure 6:**
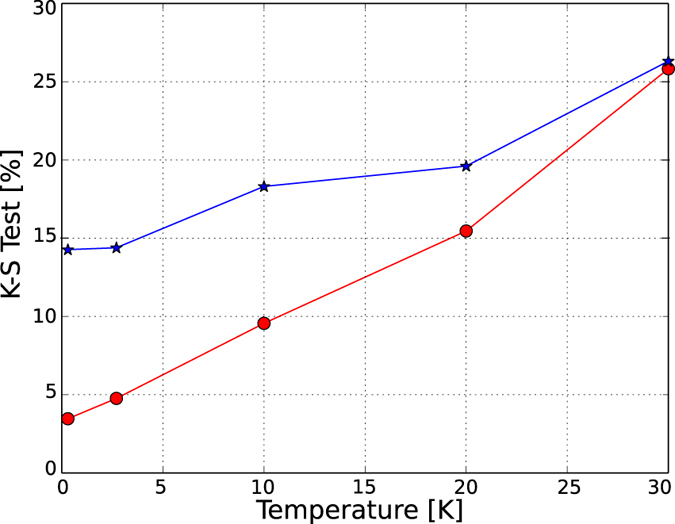
Results of the Kolmogorov-Smirnov test comparing the estimated probability functions with the theoretical GOE distribution for the N (red 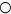) and P (blue 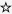) regions as functions of temperature.

**Figure 7:**
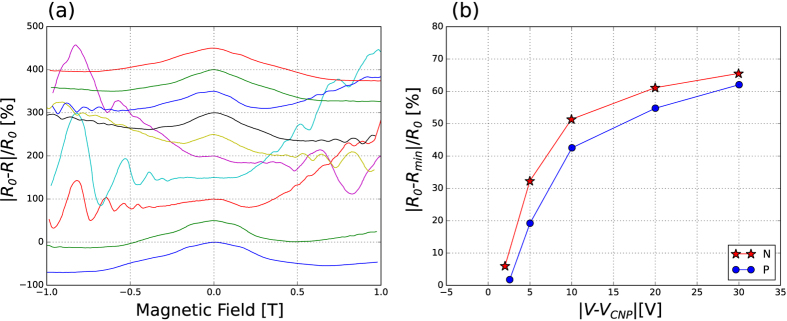
(**a**) Change in resistance with respect to the resistance at zero magnetic field (*R*_0_) for different distances from the charge neutrality point (*V*_*CNP*_). From top the bottom: −30.00 V, −20.00 V, −5.00 V, −2.55 V, 2.12 V, 5.00 V, 8.00 V, 14.16 V, 20.00 V and 30.00 V. Offsets were added for better visualization. (**b**) Maximum change in resistance for the central peak of the magnetoresistance curves for the P (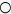) and N (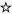) regions as a function of the *V*_*CNP*_.

The sample was fabricated by a modified dry-transfer process^29^ wherein a flake of monolayer graphene is sandwiched between two sheets of *h*-BN on top of 300 nm of SiO_2_. Gray value analysis was used to identify the thickness of the graphene flake^30^,^31^. The encapsulation technique produces samples of graphene with high carrier mobility. Four-terminal electrodes (Ti/Au) are defined by optical lithography and deposited on the corners of the graphene flake as shown in Fig. 1. The separation between the voltage probes is 2 *μ*m. The whole structure is annealed at 523 K for one hour in a reducing atmosphere to activate the contacts.

### Transport Measurements

Four-point probe quasistatic measurements were performed using a lock-in amplifier operating with an excitation current of 10 nA and a frequency of 17 Hz. The sample was in vacuum in a cryostat and the magnetic field of a superconducting magnet was applied perpendicular to the sample.

### Statistics

Recurrence plots were created using the generating function:


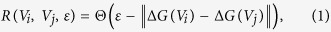


where *θ* is the Heaviside function and *ε* is a threshold value. The correlation sum was computed from this equation as


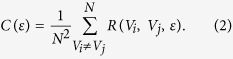


The correlation coefficient has a power-law dependence on *ε* given by *C*(*ε*) ∝ *ε*^*ν*^. The threshold *ε* used for all further calculations involving equation (1) was set to 10^−1/*ν*^, where *ν* was around 0.097.

#### Determinism

Determinism is a metric for complexity used in dynamical systems which assumes the value 0 for purely random signals and 1 for perfectly periodic signals. This metric is calculated as:


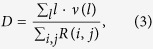


where *l* is the length of a diagonal line, and *ν*(*l*) is the frequency distribution of a diagonal line with length *l* in the recurrence plot.

#### Topology

Higushi algorithm was used to study the topology of the fluctuations^32^. The algorithm consists basically in finding the average curve length taken at different scaling coefficients *k*. This gives a power law of the form:


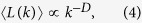


where D is related to the Hausdorff dimension *H* through *H* = 2−*D*.

#### Probability Density Function

The maxima of the fluctuations were found by taking the derivative of the smoothed fluctuations (using a 200 mV window). The differences of the positions *s* were then computed and normalized by its mean. The probability density function was estimated with the Parzen-Rosenblatt window method^33^,^34^ with a Gaussian kernel and a bandwidth of 1.06 · *σn*^−1/5^, where *σ* is the standard deviation of the samples and *n* is the sample size.

#### Linear Regressions

Ordinary least squares regression was used for linear fittings. For small populations, such as in the calculation of activation energies, Tikhonov regularization was used with a regularization parameter determined *ad hoc*.

### Gaussian Ensembles

The Gaussian ensembles are given by:


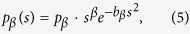


where *s* is the normalized spacing between eigenvalues, *p*_*β*_ and *b*_*β*_ are normalizing constants and *β* is 1 for the GUE, 2 for the GOE, and 4 for the GSE.

The energy values used to calculate *s* were estimated from the transconductance measurements as:


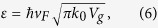


where *k*_0_ is 6.77 × 10^14^, *ν*_*F*_ is the Fermi velocity and *V*_*g*_ is a voltage point in the transconductance curve where a peak occurs.

### Kolmogorov-Smirnov

The Kolmogorov-Smirnov statistic was calculated as:


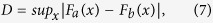


where *sup*_*x*_ is the supremum of a set and *F*_*c*_ is a cumulative distribution function. The statistic is zero for equal distributions.

## Additional Information

**How to cite this article**: da Cunha, C. R. *et al*. Conductance fluctuations in high mobility monolayer graphene: Nonergodicity, lack of determinism and chaotic behavior. *Sci. Rep.* **6**, 33118; doi: 10.1038/srep33118 (2016).

## Supplementary Material

Supplementary Information
