# Supplementary material for: Conductance fluctuations in high mobility monolayer graphene: Nonergodicity, lack of determinism and chaotic behavior
Source: Sci Rep. 2016 Sep 9;6:33118. doi: 10.1038/srep33118 (PMC5016828; doi:10.1038/srep33118)
Supplement: Supplementary Information [file srep33118-s1.pdf]

Conductance fluctuations in high mobility monolayer graphene: Non-ergodicity, lack of determinism and chaotic behavior

C. R. da Cunha, M. Mineharu, M. Matsunaga, N. Matsumoto, C. Chuang, Y. Ochiai, G.-H. Kim, K. Watanabe, T. Taniguchi, D. K. Ferry, N. Aoki

Supplementary Information

Mobility

The mobility of the sample can be estimated from its conductance curve by considering that the latter is given by:

$$\sigma = nq\mu, \tag{1}$$

where n is the carrier concentration, μ is the mobility and q is the fundamental charge.

Considering a parallel plate capacitor mode where the graphene flake is sandwiched between BN sheets, the carrier density is given by:

$$nq = CV, \tag{2}$$

where C is the capacitance, which in our case is 1.09×10^{-8} F/cm^2.

Combining both equations one can find the mobility as:

$$\mu = \frac{1}{C}\frac{\sigma}{V}. \tag{3}$$

The conductance and the mobility of the graphene sample as a function of the back gate voltage are shown in Figure S1. The maximum mobility for the P region is 101263 cm^2/V·s and is 3.0 V away from the charge neutrality point. The maximum mobility for the N region is 98140 cm^2/V·s and is 2.4 V away from the charge neutrality point.

Quantum Capacitance

The quantum capacitance is given by:[1]

$$C_q = \frac{g_v m^* e^2}{\pi \hbar^2}, \tag{4}$$

where g_v is the valley degeneracy factor, m* is the effective mass and e is the electron charge. In our case, C$_q$ is approximately 10 μF/cm^2, which is three orders of magnitude larger than the expected capacitance of SiO_2 (0.01 μF/cm^2). The effect of the quantum capacitance is thus negligible for this sample.

Berry Phase

From the Lifshitz-Onsager quantization rule[2], the Shubnikov-de Haas oscillations in graphene have an oscillatory component given by:

$$\Delta\rho_{xx} \propto cos\left[2\pi\left(\frac{F}{B} + 1/2 + \beta\right)\right], \tag{5}$$

where F is the oscillation frequency, and β is the Berry phase. The argument $i(B) = F/B + 1/2 + \beta$ returns integer values for values of B corresponding to maxima in the oscillations. Thus, by plotting these integer values as a function of $1/B$ it is possible to find the Berry phase from the intercept (i_0) as $\beta = i_0 - 1/2$. A simpler way of obtaining the Berry phase is by plotting the Landau indices corresponding to the maxima of the resistance oscillations shifted by $1/2$ and then directly finding the intercept as shown in Fig. S2. Values of $\beta = \pm 1/2$ indicate the presence of Dirac fermions, whereas $\beta = 0$ correspond to the trivial case.

The data shown in Fig.S2 depict Berry phases close to $1/2$ to most gate voltages, corresponding to the presence of Dirac fermions in our samples.

[1] S. Luryi, *Appl. Phys. Lett.* **52** (1988) 501.
[2] L. Onsager, *Phil. Mag.***43** (1952) 1006, and I. M. Lifhitz & A. M. Kosevich, *Sov. Phys. JETP* **2** (1956) 636.

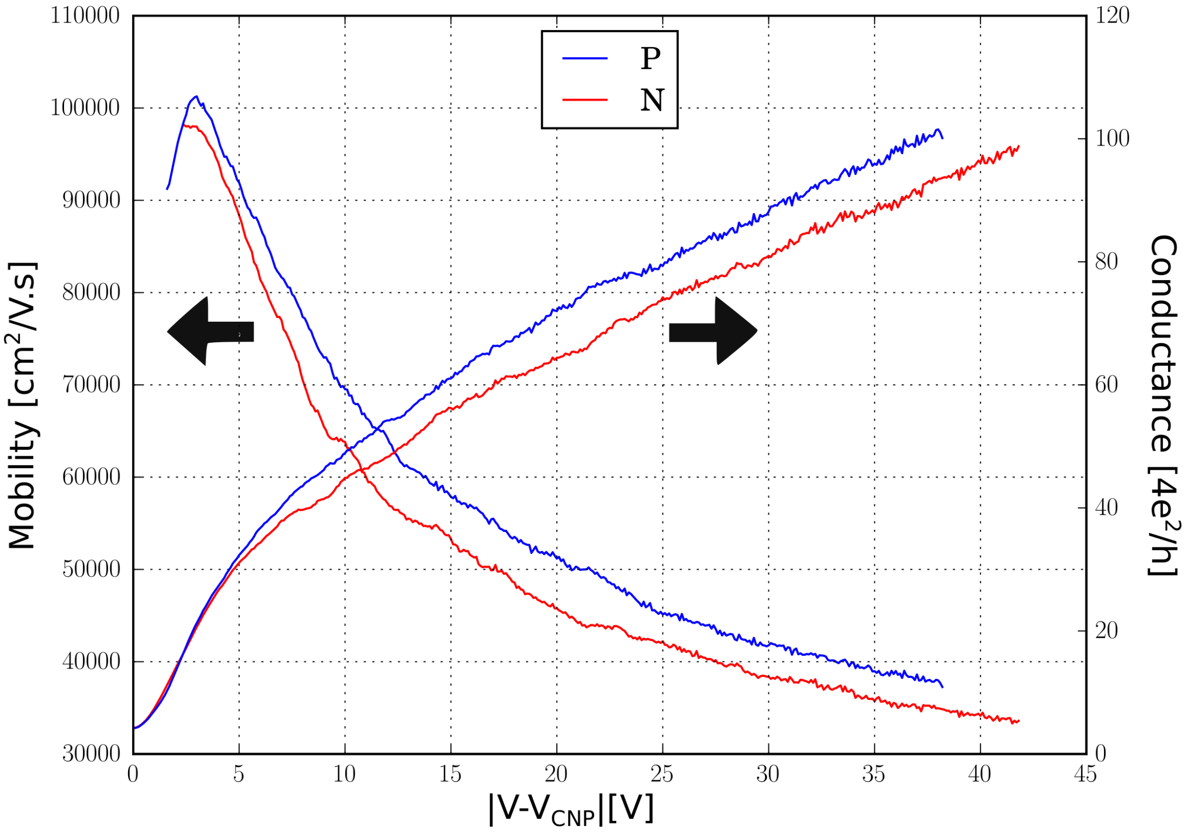

Figure S1. Conductance and mobility of the graphene sample as a function of the distance from the charge neutrality point $|V - V_{CNP}|$.

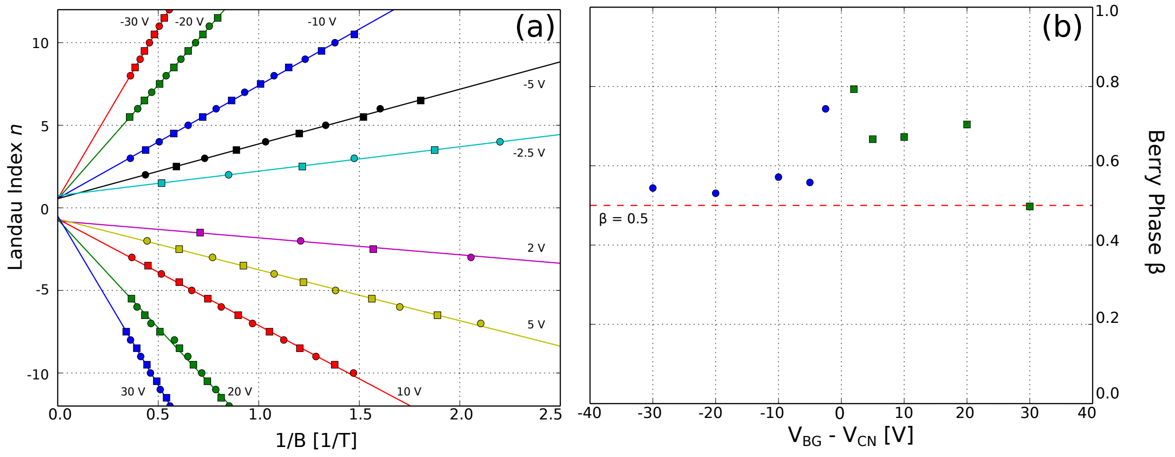

Figure S2. a) Fan diagram obtained from the Shubnikov-de Haas oscillations at different gate voltages. Circles indicate the minima of the resistance oscillations corresponding to integer Landau indices n, whereas squares indicate the maxima of the oscillations corresponding to $n \pm 1/2$. Solid lines are linear fittings. b) The Berry phase β at different gate voltages. Circles correspond to Berry's phases obtained for p-type carriers and squares correspond to phases obtained for n-type carriers. The charge neutrality point V_{CN} is subtracted from the gate voltage in both figures.

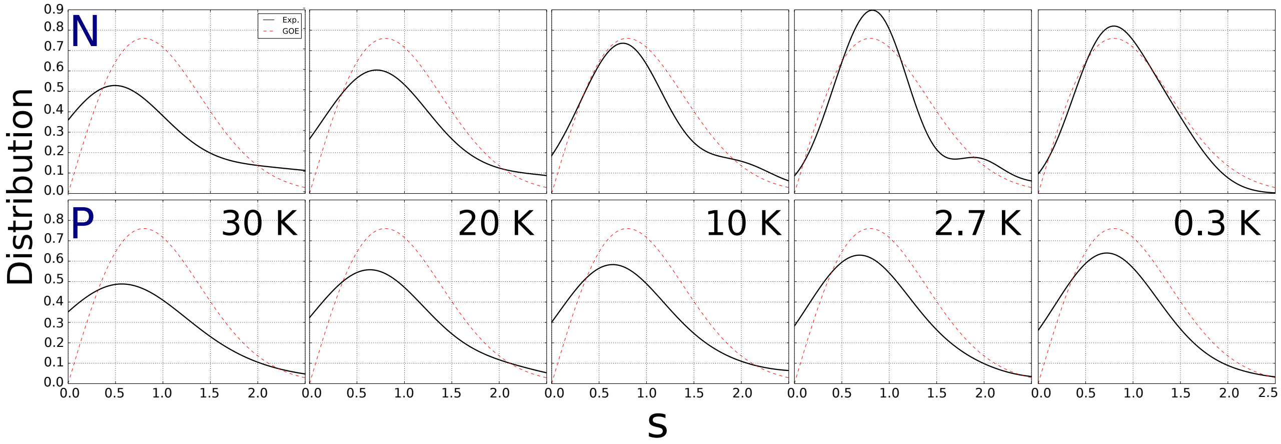

Figure S3. Estimated probability density functions (solid curves) for the P (lower row) and N regions (upper row) for different temperatures (columns) as a function of the spacing '*s*'. The theoretical GOE (dashed curves) density function is also shown.
